# Plasma Neurofilament Light Chain May Be a Biomarker for the Inverse Association Between Cancers and Neurodegenerative Diseases

**DOI:** 10.3389/fnagi.2020.00010

**Published:** 2020-01-29

**Authors:** Shunjie Liu, Zhenze Huang, Lijin Zhang, Junhao Pan, Qingfeng Lei, Yangyang Meng, Zhong Li

**Affiliations:** ^1^Department of Neurology, The Sixth Affiliated Hospital of Sun Yat-sen University, Guangzhou, China; ^2^Department of General Surgery, The Sixth Affiliated Hospital of Sun Yat-sen University, Guangzhou, China; ^3^Department of Psychology, Sun Yat-sen University, Guangzhou, China; ^4^Shenzhen Research Institute of Sun Yat-sen University, Shenzhen, China; ^5^Guangdong Provincial Key Laboratory of Brain Function and Disease, Guangzhou, China

**Keywords:** neurofilament light chain, gastric cancer, Alzheimer’s disease, biomarker, inverse association

## Abstract

An inverse association may exist between cancers and neurodegenerative diseases, although convenient biomarkers for verifying this inverse association are lacking. Plasma neurofilament light chain (NfL) is a novel biomarker for neurodegenerative diseases, such as Alzheimer’s disease (AD), but it has not been measured in patients with cancers, such as gastric cancer (GC). We aimed to explore whether plasma NfL could be a biomarker for GC and AD and whether an inverse association of NfL exists between GC and AD. In this study, plasma NfL levels of 60 normal controls (NC), 91 GC subjects, and 74 AD subjects were measured by a highly sensitive single-molecule array assay. We found that GC subjects expressed lower plasma NfL levels but AD subjects expressed higher plasma NfL levels than NCs. After controlling for confounding factors, plasma NfL levels in the GC group were associated with serum tumor marker levels, and plasma NfL levels in the AD group were associated with cognitive performance and cerebrospinal fluid (CSF) pathological marker levels. Across the entire cohort, plasma NfL levels were associated with cognitive performance, CSF pathological marker levels and serum tumor marker levels. These results suggest thatplasma NfL may be a potential biomarker for GC and AD and may be convenient for evaluating the inverse association between cancers and neurodegenerative diseases.

## Introduction

With the agingworldwide population, the prevalence of cancers and neurodegenerative diseases is rapidly rising (Wimo et al., 2013; Bray et al., 2018). These two types of diseases have both become key focuses of research because oftheir high mortality rate and lack of efficient treatments at the highly progressed stage. Thus, early diagnosis of cancers and neurodegenerative diseases and preventing their progression are of great significance. Regretfully, few convenient and accurate biomarkers for diagnosis are currently available in the clinic.

An inverse association may exist between cancers and chronic neurodegenerative diseases. On the one hand, epidemiological data have shown pronounced shifts in the incidence and mortality between cancers and neurodegenerative diseases during aging (Harding et al., 2012); the occurrence of cancers in patients with common neurodegenerative diseases and the occurrence of neurodegenerative diseases in persons with cancer are both reduced (West et al., 2005; Driver et al., 2012; Musicco et al., 2013). On the other hand, some transcriptomics studies have observed transcriptional alterations in neurodegenerative diseases in the opposite direction to the alteration observed in cancers (Klus et al., 2015; Aramillo Irizar et al., 2018). However, there is still a lack of objective and convenient biomarkers to verify this potential inverse association.

Alzheimer’s disease (AD) is the most common chronic neurodegenerative disease, characterized by a gradual decline in cognitive function that leads to problems in daily life (Gutierrez and Vitorica, 2018). The common diagnostic methods for AD have some disadvantages: ordinary brain magnetic resonance imaging (MRI)can only minimally reveal brain atrophy at the early stage (Chandra et al., 2019), and examining pathological biomarker levels (i.e.,amyloid β-protein and tau protein) in the cerebrospinal fluid (CSF) through lumbar puncture is difficult to routinely apply, given the invasivity of the procedure (Liu et al., 2019).

Gastric cancer (GC) remains a prominent cancer worldwide, as the fifth most frequently diagnosed cancer and the third leading cause of cancer death (Bray et al., 2018). Diagnostic methods for GC in present clinical practice also have deficiencies: histopathological analysis, although considered the established gold standard for detection, is too complex and invasive to be used as a screening test (Hunt et al., 2015); classical serum tumor markers, such as markers in the carcinoembryonic antigen (CEA) and carbohydrate antigen (CA) families, have relatively low specificity and sensitivity and are easily affected by other factors (Abbas et al., 2017); new biomarkers, such as circulating microRNAs, are still far from clinical use (Faghihloo et al., 2016; Mirzaei et al., 2016; Jamali et al., 2018; Simonian et al., 2018). Therefore, in the present study, we chose AD and GC as a representative neurodegenerative disease and cancer, respectively, to explore potentially convenient and accurate biomarkers for their diagnoses.

Neurofilament light chain (NfL), which is the most abundant intermediate filament protein in myelinated subcortical axons, plays an important role in the assembly and maintenance of the axonal cytoskeleton (Alirezaei et al., 2019). Disruption of axons or death of neuronsreleases NfL into the CSF, and NfL ultimately becomes widely distributed in both the CSF and the blood due to its low molecular weight and high solubility (Fuchs and Cleveland, 1998). Therefore, patients with various chronic neurodegenerative diseases, which are characterized by similar pathological changes of massive neuronaldeath and axonal disruption, have been reported to express abnormally increased NfL in their blood (Khalil et al., 2018; Alirezaei et al., 2019). Particularly, some studies have noted that AD patients also express much higher NfL levels in their blood than healthy elderly subjects (Zhou et al., 2017; Preische et al., 2019; Shi et al., 2019). Thus, peripheral NfL may be a biomarker for AD.

Patients with cancer have a reduced risk of common neurodegenerative diseases (West et al., 2005; Driver et al., 2012). This phenomenon may be due to the inverse activities of thetumor suppressor gene p53, the enzyme Pin1 and the Wnt signaling pathway between patients with cancers and patients with neurodegenerative diseases, as alterations of these factors affect the cellcycle of cells throughout the whole body (Behrens et al., 2009). Accordingly, apoptotic cell death of neurons in the brains of patients with cancers may also be suppressed by the above factors, leading to less NfL release from axons and lower NfL levels in the blood. Therefore, we speculated that reduced peripheral NfL levels may be a biomarker for GC.

The aims of thisstudy were as follows: (a) to probe differences in plasma NfL levels between AD patients and healthy elderly subjects to verify the conclusions put forth by previous studies; (b) to probe differences in plasma NfL levels between GC patients and healthy elderly subjects to explore whether NfL could be a potential biomarker for diagnosingGC; (c) to probe differences in plasma NfL levels between AD patients and GC patients to explore whether NfL could be a convenient biomarker for detecting the inverse association between cancers and chronic neurodegenerative diseases.

## Materials and Methods

### Subjects

Our study enrolled 60 normal controls (NC), 93 GC subjects and 75 AD subjects. Among them, the plasma NfL levels of 2 GC subjects and 1 AD subject could not be measured due to certain limitations of our experimental facilities. Therefore, the data of the remaining 60 NCs, 91 GC subjects, and 74 AD subjects were used for statistical analysis.

Subjects with AD and GC were consecutively selected among the patients evaluated at the Department of Neurology and the Department of General Surgery, The Sixth Affiliated Hospital of Sun Yat-sen University. NCs were enrolled from the same community and matched for sex and age.

All subjects diagnosed with AD fulfilled the following criteria: (a) age ≥ 65 years; (b) met the DSM-V 2013 criteria and the National Institute on Aging-Alzheimer’s Association (NIA-AA) 2011 criteria for dementia (McKhann et al., 2011; American Psychiatric Association, 2013); (c) had Mini-Mental State Examination (MMSE) scores below 24 and global Clinical Dementia Rating (CDR) scores between 0.5 and 2; and (d) absence of common cancers by medical examinations;

All subjects diagnosed with GC fulfilled the following criteria: (a) age ≥ 65 years; (b) histopathologically confirmed primary cancer of the stomach; (c) stagesI–III disease according to the American Joint Committee on Cancer (AJCC) 7th edition (Edge et al., 2010); (d)no prior treatments; (e) absence of other cancers according to medical examinations; (f) absence of possible paraneoplastic neurological syndromes or brain metastasesbased on the established criteria (Graus et al., 2004; Videtic et al., 2009); (g) no reports of experiencing memory loss; and (h) CDR scores of 0 and MMSE scores between 24 and 30.

The NCs fulfilled the following criteria: (a) age ≥ 65 years; (b) no reports of experiencing memory loss; (c) CDR scores of 0 and MMSE scores between 24 and 30; and (d) absence of common cancers according to medical examinations.

The exclusion criteria for all subjects included the presence of other neurological, psychiatric (i.e., schizophrenia, depression or anxiety), cerebrovascular (i.e., stroke, transient ischemic attack or intracranial haemorrhage), medical or metabolic disorders that might interfere with our study.

### Plasma Collection and Storage

Blood was collected early in the morning. Blood (4 mL) was drawn from the median cubital vein and sent to the Department of Laboratory for serum tumormarker analysis (the upper limits of normal were defined as 5 ng/mL for CEA, 37 U/mL for CA 19-9 and 35 U/mL for CA 125) by using chemiluminescence assays (Beckman Coulter; Fullerton, CA, United States). Another2 mL sample was collected in standard polypropylene EDTA test tubes (Improve Medical; Guangzhou, China). The EDTA test tubes were left upright at room temperature for 10 min before centrifugation. After centrifuging (2000 *g*, + 4°C, 10 min) the tubes, the supernatant liquid (i.e.,plasma) was transferred with a disposable transfer pipette to a 1.5 mL EP tube (JET BIOFUL; Guangzhou, China) and immediately stored at −80°C until analysis.

### Cerebrospinal Fluid Collection and Storage

A total of 32 NC, 14 GC and 49 AD subjects underwent lumbar puncture to obtain cerebrospinal fluid (CSF). The processes were performed immediately after venepuncture. Lumbar punctures were performed at the L3/L4 or L4/L5 intervertebral region. The first milliliter of CSF sample was discarded, 2 mL was sent to the Department of Laboratory for routine biochemical testingand cell analysis, and 4 mL was collected in 5 mL plain polypropylene test tubes (JET BIOFUL; Guangzhou, China). After centrifuging (2000 *g*, + 4°C, 10 min) the tubes, the supernatant liquid was immediately stored in 5 mL plain polypropylene test tubes (JET BIOFUL; Guangzhou, China) at −80°C until analysis.

### Plasma NfLMeasurements

As described in Preische et al. (2019), plasma NfLwas measured by using a highly sensitive single-molecule array assayon the Quanterix Simoa HD-1 Platform (Simoa; Quanterix, Lexington, MA, United States). Samples were measured at a 1:4 dilution by using Tris-buffered saline (Abcam; Cambridge, United Kingdom). Batch-prepared controls and calibrators (bovine lyophilizedNfL) (Uman Diagnostics; Sweden) ranging from 0 to 10,000 pg/mL were stored at −80°C until analysis. Measurements were made by experienced technicians who were blinded to the clinical data.

### CSF Core Pathological Marker Measurements

Thecore pathological markers, including amyloid β-protein 1–42 (Aβ_1__–__42_), total tau (t-tau) and phosphorylated tau 181 (p-tau_181_), in the CSF of AD patients were measured by using sandwich enzyme-linked immunosorbent assay (ELISA) kits (CUSABIO; Baltimore, MD, United Kingdom) according to the manufacturer’s instructions. The interplate and intraplate coefficients of variation of all ELISA kits were both less than 8%. The samples for ELISA were run in a randomized, blinded manner. Every sample was duplicated 3 times per plate in a total of 3 plates, and the mean of the 9 optical density values was taken as the result of the sample.

### Statistical Analysis

#### Demographics and Clinical Characteristics Analysis

The Shapiro-Wilk test was used to examine the normality of frequentist statistics. If the data fit a normal distribution, the three groups were compared by one-way analysis of variance (ANOVA), and *post hoc* comparisons were carried out by least significant difference (LSD) *t*-tests. If the data fit a non-normal distribution, the groups were compared by the Kruskal–Wallis test, and *post hoc* comparisons were carried out by the Mann–Whitney *U-*test with the *p*-values corrected according to the Bonferroni method. Categorical variables were compared using the chi-square test.

#### NfL Level Analysis

According to the Shapiro-Wilk test, the NfL level data were normally distributed. Therefore, the results of NfL levels were compared by one-way ANOVA, and *post hoc* comparisons were carried out by unpaired LSD *t*-tests if any significant difference was found.

#### Correlation Analysis

According to the Shapiro-Wilk test, the variables of age, MMSE scores and Montreal Cognitive Assessment (MoCA) scores were normally distributed. Therefore, correlations among these variables and NfL levels were calculated using Pearson’s correlation test. CSF Aβ_1__–__42_, t-tau, and p-tau_181_levels and serum CEA, CA 19-9, and CA 125 levels were non-normally distributed. Therefore, correlations among these variables and NfL levels were calculated using Spearman’s rank correlation test.

#### Multiple Linear Regression Analysis

Multiple linear regression was applied to examine the associations among cognitive ability, CSF pathological markers, serum tumor markers and plasma NfL levels. Variables that had significant correlations with plasma NfL levels were selected for inclusion in the multiple linear regression models. The models were adjusted for age, gender and education. A variance inflation factor (VIF) of 10 was determined as acut-off value for detecting multicollinearity.

Statistical significance was set at *p* ≤ 0.05. The IBM Statistical Package for the Social Sciences version 21 (IBM SPSS Statistics for Windows, IBM Corp., Armonk, NY, United States) was used for the analyses.

## Results

### Demographics and Clinical Characteristics

As reported in Table 1, there were no significant differences in age, gender ratio, years of education, HAMD scores, HAMA scores or Hachinski Ischemia scores among the three groups. Significant differences were observed in MMSE scores, Montreal Cognitive Assessment (MoCA) scores, Aβ_1__–__42_levels, t-tau levels, p-tau_181_levels, CEAlevels, CA 19-9levels, and CA 125 levels.*Post hoc* tests indicated that AD subjects had lower MMSE scores, MoCA scores and Aβ_1__–__42_levels and higher t-tau levels andp-tau_181_levels than GC subjects and NCs, with no differences between GC subjects and NCs. GC subjects expressed higher CEAlevels, CA 19-9levels and CA 125 levels than AD subjects and NCs, with no differences between AD subjects and NCs.

**TABLE 1:** Subject demographics and clinical characteristics.

Variables	NC (*n* = 60)	GC (*n* = 91)	AD (*n* = 74)	*F*_(2,222__)_	*p*	NC versus GC	NC versus AD	GC versus AD
Age (years)	71.95 ± 4.74	73.13 ± 5.60	73.20 ± 5.46	1.14	–	–	–	–
Gender (m/f)	26 m/34 f	44 m/47 f	32 m/42 f	0.56 (χ^2^)	–	–	–	–
Education (years)	9.52 ± 4.35	9.56 ± 4.14	8.41 ± 3.75	1.94	–	–	–	–
MMSE	28.53 ± 1.62	28.08 ± 1.57	21.12 ± 1.99	420.74	<0.001*	–	<0.001*	<0.001*
MoCA	27.37 ± 1.59	27.40 ± 1.74	20.03 ± 1.90	433.92	< 0.001*	–	<0.001*	<0.001*
HAMD	4.58 ± 1.79	4.88 ± 1.90	4.43 ± 1.99	1.18	–	–	–	–
HAMA	4.60 ± 1.53	4.47 ± 1.72	4.12 ± 1.81	1.47	–	–	–	–
Hachinski Ischemia	1.52 ± 1.16	1.63 ± 1.19	1.70 ± 1.27	0.39	–	–	–	–
Aβ_1__–__42_ (pg/mL)	619.53 (401.36 − 887.62)	641.48 (429.62 − 849.80)	511.06 (286.17 − 761.14)	14.67 (*H*)	0.001*	–	0.006*^#^	0.007*^#^
t-tau (pg/mL)	282.62 (159.64 − 460.15)	277.79 (192.61 − 443.08)	414.93 (257.05 − 612.75)	37.44 (*H*)	<0.001*	–	<0.001*^#^	<0.001*^#^
p-tau_181_ (pg/mL)	35.97 (19.46 − 54.18)	32.43 (20.88 − 58.14)	57.37 (28.36 − 94.17)	40.57 (*H*)	<0.001*	–	<0.001*^#^	<0.001*^#^
CEA (ng/mL)	1.79 (0.06 − 8.34)	4.81 (0.12 − 113.18)	1.66 (0.03 − 6.16)	41.97 (*H*)	<0.001*	<0.001*^#^	–	<0.001*^#^
CA19-9 (U/mL)	10.29 (0.41 − 34.20)	24.96 (0.94 − 2842.10)	9.06 (0.27 − 29.78)	59.94 (*H*)	<0.001*	<0.001*^#^	–	<0.001*^#^
CA125 (U/mL)	7.89 (1.09 − 33.51)	16.14 (0.92 − 482.90)	9.65 (0.63 − 41.84)	20.03 (*H*)	< 0.001*	<0.001*^#^	–	0.02*^#^

*Normally distributed data are reported as the mean ± standard deviation (SD), and non-normally distributed data are reported as the median (min-max).* *Significance at p ≤ 0.05, ^#^p with the Bonferroni correction. The symbol “–” indicates no significant difference. NC, normal control; GC, gastric cancer; AD, Alzheimer’s disease; MMSE, Mini-Mental State Examination; MoCA, Montreal Cognitive Assessment; HAMD, Hamilton Depression Rating Scale; HAMA, Hamilton Anxiety Scale; Aβ_1__–__42_, amyloid β-protein 1–42; t-tau, total tau; p-tau_181_, phosphorylated tau 181; CEA, carcinoembryonic antigen; CA 19-9, carbohydrate antigen 19-9; CA 125, carbohydrate antigen 125.

### Plasma NfLAnalysis

**FIGURE 1:**
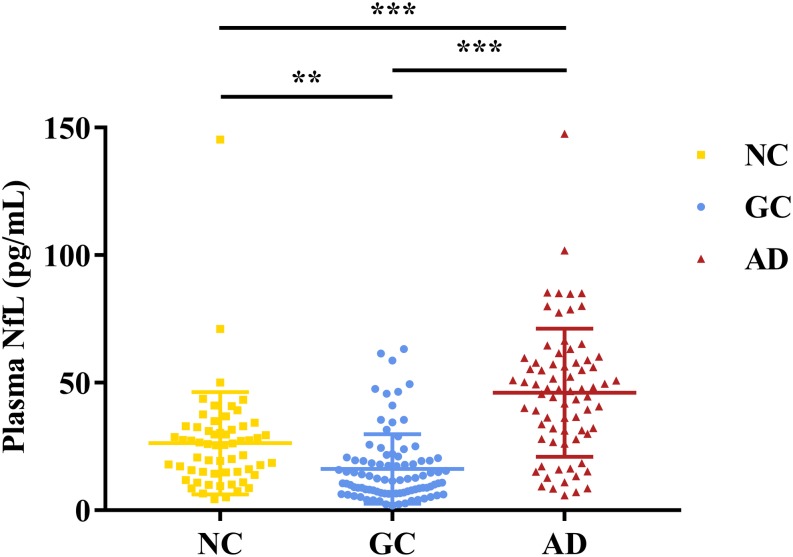
Plasma NfL levels in NCs, GC subjects and AD subjects. The y-axis showsplasma NfL levels in the different groups. Error bars indicate SD.*p<0.05, **p<0.01, ***p<0.001.NC, normal control; GC, gastric cancer; AD, Alzheimer’s disease.

As shown in Figure 1, the plasmaNfL levels of NCs (26.26 ± 20.05pg/mL), GC subjects (16.17 ± 13.66pg/mL), and AD subjects (46.07 ± 25.16pg/mL) were significantly different[*F*_(__2_,_222__)_ = 47.30, *p* < 0.001]. *Post hoc* tests indicated that AD subjects expressed higher NfL levels than NCs and GC subjects, and NCs showed higher NfL levels than GC subjects.

### Correlation Analysis

**FIGURE 2:**
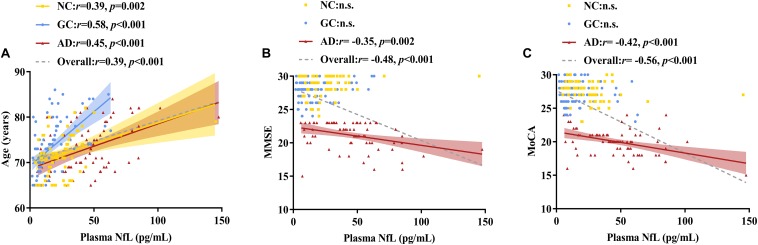
Correlations of plasma NfL levels with age, MMSE scores and MoCA scores. **(A)** Relationship between plasma NfL and age. **(B)** Relationship between plasma NfL and MMSE. **(C)** Relationship between plasma NfL and MoCA. Pearson’s correlation tests were employed. The numbers are the Pearson correlation coefficients. The symbol “n.s.” indicates no significant association. NfL, neurofilament light chain; MMSE, Mini-Mental State Examination; MoCA, Montreal Cognitive Assessment; NC, normal control; GC, gastric cancer; AD, Alzheimer’s disease.

**FIGURE 3:**
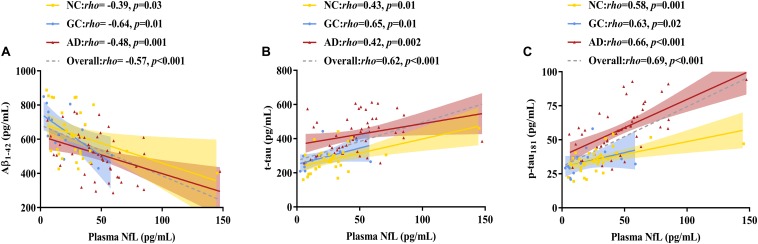
Correlations between plasma NfL levels and CSF pathological biomarker levels. **(A)** Relationship between plasma NfL and Aβ_1__–__42_. **(B)** Relationship between plasma NfL and t-tau. **(C)** Relationship between plasma NfL and p-tau_181_. Spearman’s rank correlation test was employed. The numbers are the Spearman correlation coefficients. The symbol “n.s.” indicates no significant association. NfL, neurofilament light chain; Aβ_1__–__42_, amyloid β-protein 1–42; t-tau, total tau; p-tau_181_, phosphorylated tau 181; NC, normal control; GC, gastric cancer; AD, Alzheimer’s disease.

As shown in Figures 2, 3 and Table 2, plasma NfL levels were positively correlated with age, t-tau and p-tau_181_ levels and negatively correlated with Aβ_1__–__42_ levels within each group and across the entire cohort. Plasma NfL levels were negatively correlated with MMSE and MoCA scores within the AD group and across the entire cohort. Plasma NfL levels were negatively correlated with CEA, CA 19-9 and CA 125 levels within the GC group and across the entire cohort.

**TABLE 2:** Correlations between plasma NfL levels and serum tumor marker levels.

	NfL			
Group	NC	GC	AD	Overall
CEA	–	−0.44* (−0.63, −0.24)	–	−0.39* (−0.51, −0.28)
CA 19-9	–	−0.35* (−0.56, −0.14)	–	−0.46* (−0.56, −0.33)
CA 125	–	−0.52* (−0.67, −0.34)	–	−0.23* (−0.35, −0.09)

*Numbers are the Spearman correlation coefficients with 95% confidence intervals.* *Significance at p ≤ 0.05. The symbol “–” indicates no significant association. NfL, neurofilament light chain; NC, normal control; GC, gastric cancer; AD, Alzheimer’s disease; CEA, carcinoembryonic antigen; CA 19-9, carbohydrate antigen 19-9; CA 125, carbohydrate antigen 125.

### Multiple Linear Regression Analysis

No multicollinearity was detected in any of the models. As reported in Table 3, plasma NfL levels were not significantly associated with CSF pathological marker levels within the NC or GC group. PlasmaNfL levels were negatively associated with CEA levels and CA 125 levels in the GC group. Plasma NfL levels were negatively associated with MMSE scores, MoCA scores and Aβ_1__–__42_ levels and positively associatedp-tau_181_ levels within the AD group. Plasma NfL levels were negatively associated with MoCA scores, Aβ_1__–__42_ levels, CEA levels and CA 125 levels and positively associatedp-tau_181_ levels across the entire cohort.

**TABLE 3:** Multiple linear regression analysis: predictors of plasma NfL levels.

	NC		GC		AD		Overall	
	β	*p*	β	*p*	β	*p*	β	*p*
MMSE					–3.34	0.01*	–0.40	0.51
MoCA					–4.73	< 0.001*	–2.76	< 0.001*
Aβ_1__–__42_	–0.03	0.17	–0.07	0.27	–0.05	0.03*	–0.05	0.005*
t-tau	0.15	0.16	0.02	0.81	0.03	0.28	0.41	0.08
p-tau_181_	0.71	0.13	0.38	0.42	0.77	< 0.001*	0.68	< 0.001*
CEA			–0.14	0.02*			–0.18	0.02*
CA 19-9			–0.002	0.64			–0.002	0.72
CA 125			–0.04	0.005*			–0.05	0.01*

*β represents the partial regression coefficient.* *Significance at p ≤ 0.05. NC, normal control; GC, gastric cancer; AD, Alzheimer’s disease; MMSE, Mini-Mental State Examination; MoCA, Montreal Cognitive Assessment; Aβ_1__–__42_, amyloid β-protein 1–42; t-tau, total tau; p-tau_181_, phosphorylated tau 181; CEA, carcinoembryonic antigen; CA 19-9, carbohydrate antigen 19-9; CA 125, carbohydrate antigen 125.

## Discussion

The key aim of the present study was to compare plasma NfL levels among NCs, GC patients and AD patients. On the one hand, consistent with previous studies (Zhou et al., 2017; Lewczuk et al., 2018; Preische et al., 2019; Shi et al., 2019), we found that AD patients expressed significantly higher plasma NfL levels than NCs. On the other hand, to the best of our knowledge, we are the first to reveal the trends in plasma NfL levels in GC patients and found that GC patients expressed significantly lower plasma NfL levels than NCs.

The inverse trends of three factorsthatregulate cell survival may contribute to the inverse association between cancers and neurodegenerative diseases. In a wide range of organisms, over-expression of the tumor suppressor gene p53, as well as inactivation of the enzyme Pin1 and the Wnt signaling pathway, lead cells to a prone-to-death state (neurodegenerative disease phenotype); lowexpression of the p53 gene, as well as activation of the enzyme Pin1 and the Wnt signaling pathway, lead cells to a prone-to-survival state (cancer phenotype) (Yaffe et al., 1997; Coombs et al., 2008; Murray-Zmijewski et al., 2008; Behrens et al., 2009). That is, neurons in the AD patients enrolled in our study may be in a prone-to-death state, leading to more NfL release from the axonal membrane and higher plasma NfL levels; neurons in the GC patients enrolled in our study may be in a prone-to-survival state, leading to less NfL release from the axonal membrane and lower plasma NfL levels. These alterations in cell survival mechanisms could theoretically explain the significant differences in plasma NfL levels observed in our study.

Through the correlation analysis, we discovered that plasma NfL levels were positively associated with age, in agreement with the results of previous studies (Zhou et al., 2017; Lewczuk et al., 2018). Unsurprisingly, with aging, more neurons senescence and undergo apoptosis, causing greater NfL release and higher plasma NfL levels. Our results also revealed that CSF pathological marker levels were significantly correlated with plasma NfL levels within the NC and GC groups, however, these associations were no longer statistically significant after controlling for other confounding factors in the multiple linear regression models. This non-conformity suggested that CSF pathological markers play a limited role in plasma NfL levels, which is probably due to floor effects of pathological changes in subjects with intact cognitive function (i.e., most NCs and GC subjects did not have obvious Aβdeposition or tau protein hyperphosphorylation, and thus, they had very similar levels of Aβ_1__–__42_, t-tau andp-tau_181_ in their CSF). The multiple linear regression analysisrevealed that CSF pathological marker levels and cognitive performance were significantly associated with plasma NfLlevels within the AD group and across the entire cohort, which was also consistent with previous studies (Zhou et al., 2017; Lewczuk et al., 2018). These associations can be easily explained: (a) higher plasma NfL levels indicate more serious neuronal loss, which leads to worse cognitive performance as revealed in neuropsychological tests; (c) lower Aβ_1__–__42_ levels and higher tau protein levels in the CSF indicate that more neurons are contaminated by Aβplaques and have abnormal tau protein in the cytoskeleton. These neurons degenerate much more easily than normal neurons (LaFerla et al., 1995; Bouge and Parmentier, 2016), causing more NfLrelease. However, we found that plasma NfL levels were not correlated with MMSE or MoCA scores within the NC and GC groups, which was probably due to ceiling effects of these two tests in subjects with intact cognitive function (i.e., most NC and GC subjects scored nearly full marks on these tests) (Zhou et al., 2017).

To our knowledge, we are the first to reveal negative associations of plasma NfL levels with serum tumor marker levels within a GC group and across the entire cohort. A review of GC suggested that higher tumor marker levels indicate larger tumors, greater serosal attack and elevated rates of metastases (Abbas et al., 2017), which may be caused by greater dysfunction of cell-cycle regulators (i.e., the p53 gene, the enzyme Pin1 and the Wnt signaling pathway, as mentioned above). Thegreater dysfunction of these three regulators may drive more neurons towarda prone-to-survival state, leading to less NfL release and lower plasma NfL levels. However, no such associations were observed within the NC and AD groups, which is not surprising due to the floor effects of serum tumor markers in subjects without malignant tumors (i.e., most NC and AD subjects have very low, even close to zero, tumormarker levels).

However, there are still some limitations of our study. First, we only examined plasma NfL levels in GC patients but not in patients with other cancers. Therefore, further studies are needed to verify whether patients with other cancers also express lower plasma NfL levels. Second, as a case-control study, the changing trend in plasma NfL levels during the progression of GC and AD could not be observed. Third, CSF NfL levels were not measured or analyzedbecause only a relatively small proportion of GC subjects gave consent for lumbar puncture, which is a disadvantage of our study. In future studies, we plan to further investigate whether GC patients also express abnormally low CSF NfL levels.

## Conclusion

In conclusion, using an ultrasensitive method, we are the first to measure plasma NfL levels in GC patients. We found that GC patients had decreased levels of plasma NfL, while AD patients had increased levels of plasma NfL. The opposite trends in plasma NfL levels between GC patients and AD patients suggest that plasma NfL could be a convenient biomarker for verifying the inverse association between cancers and chronic neurodegenerative diseases.

## Data Availability Statement

The datasets generated for this study are available on request to the corresponding author.

## Ethics Statement

The Ethical Committee of The Sixth Affiliated Hospital of Sun Yat-sen University approved this study and all subjects gave their written informed consent in accordance with established human subject research procedures expressed in Declaration of Helsinki.

## Author Contributions

SL and ZL conceived and designed the study. ZH, QL, and YM recruited the subjects and obtained clinical results. SL performed the NfL measurement. LZ and JP performed the statistical analysis. All authors wrote and revised the manuscript.

## Conflict of Interest

The authors declare that the research was conducted in the absence of any commercial or financial relationships that could be construed as a potential conflict of interest.

## Abbreviations

NfL: neurofilament light chain
AD: Alzheimer’s disease
GC: gastric cancer
NC: normal control
CSF: cerebrospinal fluid
MRI: magnetic resonance imaging
CEA: carcinoembryonic antigen
CA: carbohydrate antigen
MMSE: Mini-Mental State Examination
CDR: Clinical Dementia Rating
AJCC: American Joint Committee on Cancer
MoCA: Montreal Cognitive Assessment
HAMD: Hamilton Depression Rating Scale
HAMA: Hamilton Anxiety Scale
A β_1–42_: amyloid β-protein 1–42
t-tau: total tau
p-tau_181_: phosphorylated tau 181
ANOVA: analysis of variance
LSD: least significant difference
VIF: variance inflation factor.
